# Chemical Modification of Plasticized Lignins Using Reactive Extrusion

**DOI:** 10.3389/fchem.2019.00633

**Published:** 2019-09-18

**Authors:** Romain Milotskyi, László Szabó, Kenji Takahashi, Christophe Bliard

**Affiliations:** ^1^Institut de Chimie Moléculaire de Reims, ICMR, CNRS UMR 7312, URCA, B18, UFR SEN, Reims, France; ^2^Institute of Science and Engineering, Kanazawa University, Kanazawa, Japan

**Keywords:** plasticizing, kraft lignin, esterification, ^31^P NMR, DMSO, extrusion

## Abstract

The reaction of esterification of plasticized Kraft lignin (KL) with succinic and maleic anhydrides using reactive extrusion (REX) was studied in detail. DMSO, glycol and glycerol were found to be efficient plasticizers for lignin. The chemical structure of these new lignin esters was determined using Solid-state ^13^C CP-MAS NMR and FT-IR analysis. ^31^P NMR analysis of phosphitylated lignins showed that the aliphatic OH groups of lignin had superior reactivity under the reactive extrusion reaction conditions. The formation of monoesters was confirmed by HSQC NMR spectroscopy. Molecular weight changes after extrusion process were studied using GPC/SEC chromatography. Thermal properties of these polymers were assessed by TGA analysis. The results were compared to lignin esters modified in classical batch conditions. These results show that REX can be used as a new fast, solvent free, and continuous process for lignin valorization.

## Introduction

Lignin constitutes one of the main chemical families in plant biomass (Gandini, 2011). Lignins are a polymeric form of variously methoxy-substituted phenylpropane monomeric units. Due to its general abundance (up to 20–30% in hardwood) and its slow biodegradability, lignin is likely to be one of the most abundant biopolymers on earth (Smolarski, 2012). Surprisingly apart from energy production in paper factories lignin finds very few industrial applications (Zakzeski et al., 2010). Production of high value added products such as vanillin has been obtained from lignin (Salvesen et al., 1948; Fache et al., 2016). The aromatic phenylpropane nature of lignin offers some similarity with aromatic polymers. Unfortunately, as its complex mixed structure depends on the extraction process, lignin processing is difficult to carry out and not conducive to chemical process development. Moreover, the difficulty in solubilizing and fractionating renders lignin difficult to process at the industrial level (in non-aqueous media). Various examples of lignin chemical modification have been developed in order to adapt the properties to specific applications (Glasser and Jain, 2009; Laurichesse and Avérous, 2014; Konduri et al., 2015; Kai et al., 2016; Ryohei et al., 2017; Sakai et al., 2018; Szabó et al., 2019). Modification of the aliphatic and aromatic hydroxyl groups of lignin via esterification is a typical approach (Laurichesse and Avérous, 2014). Several esterification agents have been applied to synthesize new lignin derivatives: acetic, propionic and butyric anhydrides (Fox and McDonald, 2010); maleic, succinic and phtalic anhydrides (Chen et al., 2014); palmitic and lauric acid chloride (Hult et al., 2013). Thielemans and Wool (2005) showed that in unsaturated thermosetting composites, incorporated butyrated kraft lignin plays a role in reinforcing, plasticizating, and influencing the cure kinetics of the polymerizing resin. Maleated lignin was successfully mixed with recycled polystyrene showing higher miscibility than native lignin (Schorr et al., 2015) and slightly higher thermal stability compared to native polystyrene (Lisperguer et al., 2013).

Reactive extrusion (REX) is a fast developing technique that shows high potential in producing chemically modified bio-sourced polymeric material from small to large scale (Berzin and Hu, 2004; Formela et al., 2018; Milotskyi et al., 2018). It can be applied to a wide range of highly viscous materials. Reactive extrusion itself can be considered as a green manufacturing process as compared to conventional chemical modification processes: it does not require solvents; it is very energy efficient; it allows clean reaction processes with fast kinetics, even when applied to large scales. Only a few examples of REX modified lignin can be found in the literature (Bridson et al., 2013; Fernandes et al., 2014; Luo et al., 2016). In this paper we describe the chemical modification of lignin with maleic and succinic anhydrides using REX. Both maleic and succinic anhydrides have the potential to be synthesized from biomass-based feedstocks (Bechthold et al., 2008; Li et al., 2018). Moreover, after anhydride opening and reaction with lignin, the free carboxyl group formed can be used for self-polyesterification to form biobased thermosetting polyester coatings (Scarica et al., 2018). More specifically, the main objective of this work was to intensify the process of lignin esterification and investigate the influence of reaction parameters on lignin conversion during REX. New plasticizer systems were developed for lignin processing with relatively low temperature (140°C).

## Experimental

### Materials

Two Lignins: KL pH6 (“Lignin, alkali” Aldrich 370959, insoluble in water) which will be noted KL in this work and KL pH10.5 (“Lignin, alkali,” low sulfonate content, Aldrich 471003, soluble in water) which will be noted KLS were provided by Sigma—Aldrich (St. Louis, USA). Succinic anhydride (SA) and maleic anhydride (MA) were purchased from Alfa Aesar (Karlsruhe, Germany). Glycerol, ethylene glycol and DMSO were purchased from VWR International (Fontenay-sous-Bois, France). The reactor used in this study was the “Minilab Rheomex CTW5” (RHEO S.A., Champlan, France). This apparatus is designed for compounding polymers in a thermostated heat block with a production capacity of 100 g/h. It is also designed for analyzing the rheological behavior of polymer melts on small scale. The system is based on a conical, twin-screw compounder. Two different types of screw geometry (co- and counter- rotating) can be used in order to study the reaction differences according to the mixing mode. Due to a particular design only very small sample amounts (10 g) are needed in its integrated backflow channel. In this study, the conical co-rotating screws mode was chosen over the counter-rotating one because of its superior mixing ability (Milotskyi and Bliard, 2018). The extruder was operated at a screw speed of 60 rpm in the direct and recirculation (loop) modes. The melt-mass flow-rate was determined by weighting extrudates in known time intervals.

### Synthetic Procedure

#### Preparation of Samples for Extrusion

At first, the processability of the two lignins KL and KLS in REX conditions was studied. All attempts to extrude the lignins without any added plasticizer resulted in blocking the extruder due to excessive torque value, exceeding the machine parameters. To address this problem the plasticizing of lignins by using different plasticizers: DMSO, glycerol, ethylene glycol was studied.

Each lignin was milled with a mortar and pestle and then mixed with the corresponding amount of plasticizer at room temperature. The mixture was then introduced into the extruder through the feed hopper and extruded in re-circulation mode to obtain melt viscosity information. The conditions and results of plasticizing are discussed further in this article.

#### Esterification of Lignins Using REX

To carry out the esterification reaction, 7 g of the plasticized KL was weighed and a corresponding mass of maleic or succinic anhydride calculated with respect to the dry mass of lignin (without plasticizer) was added. The mixture was introduced into the reactor preheated to the selected temperature and was then extruded using the direct mode. In order to evaluate reproducibility, each experiment was repeated at least three times except when noted. The amount of ester reagent (SA or MA) was 0.1, 0.2 and 0.3 equivalents per average phenylpropane unit (Molar mass 178 g/mol, average of the molecular weights of the three monomeric units *p*-coumaryl, coniferyl, and sinapyl alcohol). A reference sample named KL_ref_ was extruded with 25% DMSO without reagents at an extrusion temperature of 140°C and a screw speed of 60 rpm using the direct mode.

#### Purification of Reacted Extrudates

The KL was purified as follows to remove residues of free maleic or succinic acid or their anhydrides possibly present in the final mixture:

The reaction mixture of KL was crushed in a mortar and the obtained powder was purified with a method adapted from Chen et al. (2014) with some modifications: washing with distilled water and sodium bicarbonate for 24 h with agitation. The resulting powder was then recovered by filtering off the aqueous phase on Whatman filter paper. The modified lignin was dried for 24 h at room temperature in open air, and then overnight in a vacuum oven at 60°C.

#### Esterification of Lignins in Batch

One gram of kraft lignin (KL) was dissolved in 50 mL pyridine and 0.1/0.2/0.3 equivalent of acylating reagent -succinic anhydride or maleic anhydride- was added to the solution. The reaction was kept for 24 h at room temperature. The obtained product was collected after pouring the solution into 500 mL acetone, and dried in a vacuum drying oven at 70°C for 24 h.

### Specific Mechanical Energy (SME)

Specific mechanical energy (SME) is the amount of mechanical energy (work) transferred to the melted mix and dissipated as heat inside the material, expressed per mass unit of the material. SME is calculated using Equation 1 according to Li et al. (2014) as follows:

(1) $$\begin{array}{l} \text{SME }(\text{J}/\text{kg})=(\text{Screw speed }(\text{rpm})\times \text{Torque }(\text{N}\times \text{m})\times 60)/\\ \;(\text{Feed rate }(\text{kg}/\text{h})) \end{array}$$

### Elemental Analysis (C, H, S, N)

A Perkin Elmer 2400 Series II-CHNS/O Elemental Analyzer was used to determine carbon, hydrogen, nitrogen and sulfur contents by catalytic combustion. The oxygen content was estimated on the assumption that the samples contained only C, H, N, S, and O. The samples were dried in vacuum at 80°C prior to the analyses.

### Fourier Transform InfraRed Spectroscopy (FTIR)

The FTIR analysis was carried out in absorption mode on KBr pellets mixed with the powdered samples. The spectra were obtained on a Nicolet FT-IR 470 spectrophotometer (Nicolet Instrument Corporation, USA) at 4,000–400 cm^−1^ at a resolution of 4 cm^−1^. The number of accumulated spectra is 32.

Samples synthesized in batch conditions were analyzed using Thermo Fisher Scientific Nicolet iS10 (Thermo Fisher Scientific, Inc., Tokyo, Japan) spectrophotometer equipped with an attenuated total reflection (ATR) unit. The number of accumulated spectra is 64.

### Nuclear Magnetic Resonance Spectroscopy

The HSQC NMR spectra were obtained in DMSO-*d*_6_ as a solvent. The recording was tuned for a one bound coupling of 145 Hz. The number of scans was 48; the acquisition time 0.14 s and the relaxation delay 5 s. The data matrix was 256/2048 (covering 22,640 Hz in F1 and 7,100 Hz in F2).

Phosphorus-31 NMR spectrometry was used in order to quantify aliphatic and aromatic hydroxyl groups as well as carboxylic acid groups in lignins. In order to carry out the analysis it was necessary to phosphitylate the lignins with 2-chloro-4,4,5,5-tetramethyl-dioxaphospholane as the phosphitylating reagent prior to recording. This reagent was synthesized in our laboratory from pinacol and phosphorus trichloride and purified by distillation according to the method described by Zwierzak (1967) as follows. A solution of pinacol (0.2 mole) and distilled triethylamine (0.4 moles) in cyclohexane (150 mL) was added dropwise to a solution of phosphorus trichloride (0.2 mole) in cyclohexane (200 mL) at 5–10°C with efficient stirring and cooling. The mixture was kept for 1 h at room temperature and filtered. The resulting triethylamine hydrochloride crystals were filtered off and washed with cyclohexane (200 mL). Evaporation of the filtrate and careful vacuum distillation of the residue gave pure 2-chloro-4,4,5,5-tetramethyl-dioxaphospholane. The yield after distillation was 36%.

The solvent used for ^31^P NMR was a mixture of pyridine and deuterated chloroform (1.6:1 v:v) protected from moisture using 4 Å dry molecular sieve. A solution of relaxation agent was prepared by mixing 5 mg of relaxation reagent (chromium III acetylacetonate) in one milliliter of the prepared solvent. The internal standard solution was prepared with 10.85 mg/ml of cyclohexanol in prepared solvent. For this experiment, 30 mg of lignin was accurately weighed into a 1 mL volumetric flask. The sample was then dissolved in 0.5 mL of the above solvent mixture. 2-Chloro-4,4,5,5-tetramethyl-dioxaphospholane (50 μL) was then added, followed by the internal standard and the relaxation agent solution (100 μL each). Finally, the solution was filled to 1 mL with more solvent mixture. The volumetric flask was closed and stirred to ensure complete mixing. The spectra were obtained at 202.45 MHz with inverse-gated decoupling. The pulse lengths corresponded to a 30° pulse. The number of scans was 128, the acquisition time 0.55 s, the relaxation delay 12 s, the spectral widths 294 ppm, the carrier frequency 140 ppm.

Solid-state ^l3^C CP/MAS NMR spectra were obtained at 10 kHz on a ECX-500II spectrometer (JEOL Ltd., Tokyo, Japan). At least 4096 scans were averaged for each spectrum. In the case of sample “Batch KL0.3MA” the number of scans was increased to 10,000 to obtain a spectrum with high resolution. A single contact time of 2 ms, a delay of start of 1.01 s, and proton preparation pulse of 2.71 μs were used. Lignin methoxyl peak (52 ppm) was used as a reference. The DS is calculated using Equation 2 as described by Bridson et al. (2013),

(2) $$\begin{array}{r} \text{DS}={\text{I}}_{1}/\left({\text{I}}_{2}{\;}^{*}\text{ n}\right) \end{array}$$

where I_1_ is the sum of grafted maleic anhydride carbonyl (165 ppm) and CH integrals (126 ppm), I_2_ is the methoxyl integral (assuming a stoichiometric number of methoxyl groups), n is the number of maleate carbons (4). The corresponding reaction efficiency (RE) was calculated using Equation 3:

(3) $$\begin{array}{r} \text{RE}=\text{DS}/\left(\text{MA}/\text{L}\right){\;}^{*}\;100\% \end{array}$$

where DS is the degree of substitution obtained from Equation 2 and MA/L is the mole ratio of maleic anhydride (MA) to lignin C_9_ unit.

### GPC/SEC Chromatography

The molecular weight of native and modified lignins was determined by size exclusion chromatography (SEC, Prominence UFLC system, Shimadzu Co., Kyoto, Japan) based on polystyrene standards. All GPC/SEC measurements were carried out at 40°C using TSK gel α-M (Tosoh Co., Tokyo, Japan). Samples were dissolved in DMF containing 0.01M of LiBr. The final concentration was 1 mg of polymer in 1 ml of solvent. Before injection, the samples were filtered through a 0.2 μm syringe PTFE filter. The same solvent was used as eluent at the flow rate of 1.0 ml/min. RID-10A Refractive Index Detector (Shimadzu Co., Kyoto, Japan) was used in this study.

### Thermogravimetric Analysis

The TA Instruments 2950 Thermogravimetric Analyzer (TGA) 2950 was used to determine the degradation temperature of native and modified lignin samples under nitrogen flow. The samples were analyzed up to a temperature of 650°C at a rate of 10°C/min.

## Results and Discussion

### Lignins Processing

At first, the processability of the two native lignins without plasticizer was tested in the extruder within the 140–180°C temperature range. In this case, no melt behavior was observed for both lignins in the chosen range of temperatures. Three plasticizers DMSO, ethylene glycol and glycerol were then studied as potential plasticizers. Results of lignin plasticization using the different plasticizers are given in Table 1. The extrusion temperature was fixed at 140°C with a screw speed of 60 rpm during 5 min using recirculation mode.

**Table 1:** Results of lignins plasticization with different plasticizers.

**Lignin/plasticizer system**	**Torque (N × m)**	**SME (kJ/kg)**	**Melt viscosity (Pa.s)**
KL 15% DMSO	3.3	237	4.9 × 10^3^
KL 20% DMSO	2	144	1.2 × 10^3^
KL 25% DMSO	1.8	130	4 × 10^2^
KLS 20% DMSO	1.7	122	8.96 × 10^3^
KLS 20% Et Glycol	1.4	101	2.5 × 10^3^
KLS 20% Glycerol	1,5	108	2.4 × 10^3^

In the case of the sample where 15% DMSO was used, extrusion is characterized by fairly high SME values; as the maximum torque for the Minilab Rheomex CTW5 is 5.5 N × m, below 15% DMSO the extrusion was not possible on this extruder due to excessive torque values. The addition of supplementary amounts of plasticizer decreased SME and melt viscosity. The melt viscosity values for mixtures prepared with ethylene glycol and glycerol were similar. In conclusion, DMSO gave the best results in providing lignin with a good melting behavior. The two other plasticizers used in this study, polyols -ethylene glycol and glycerol-, were only tested with KLS lignin as they were previously studied for KL lignin (Bouajila et al., 2006). DMSO was then chosen as a main plasticizer as it is compatible with lignin and it has a relatively high boiling point (191°C) compatible with the extrusion conditions and does not interfere with the reagents. In addition, to our knowledge, it had not yet been studied for lignin plasticization applications.

### Esterification of Lignins by Reactive Extrusion

At first reactive extrusion of native KL (without plasticizer) with MA and SA was tested at a temperature of 175°C, without plasticizer, as described by Bridson et al. (2013). A molar ratio of 0.2 of MA and SA was used for this test. However, no fusion was observed in our case, indicating that the addition effect of the anhydrides did not improve the melting behavior of KL lignin. REX esterification of lignin was then conducted using previously plasticized KL lignin with 25% of DMSO at 140°C.

### Elemental and FTIR Analysis

**Figure 1:**
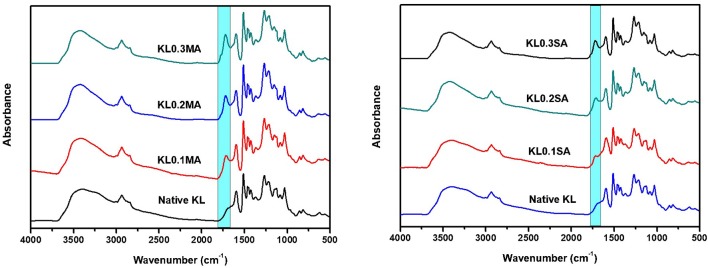
FTIR spectra of KL modified with different ratio of maleic **(Left)** and succinic **(Right)** anhydrides.

In both ester types, elemental analysis showed a decrease in the amount of analyzed carbon in all esterified products, compared to unmodified lignins. For example, the carbon content of native KL before extrusion was 63.42% (±0.11), after extrusion of KL with 0.1 maleic anhydride, it decreased to 62.01% (±0.02) and to 60.02% (±0.13) after extrusion with 0.3 MA. The decrease in carbon content was observed for KL modified with SA, from 61.88% (±0.04) for KL0.1SA to 58.86% (±0.24) for KL0.3SA. This result confirms the addition of succinic and maleic groups to the lignin, which contain a lower proportion of carbon. FTIR results show that in both cases, a band of carbonyl groups is present at 1722 cm^−1^ for 0.1, 0.2, and 0.3 MA and SA, the intensity of this band increases with the quantity of anhydride added (Figure 1). All these results confirm the substitution of OH groups in lignins and their transformation into esters. FTIR spectra of samples synthesized in batch are presented in Supporting Information (Figures S1, S2).

### HSQC NMR Analysis

**Figure 2:**
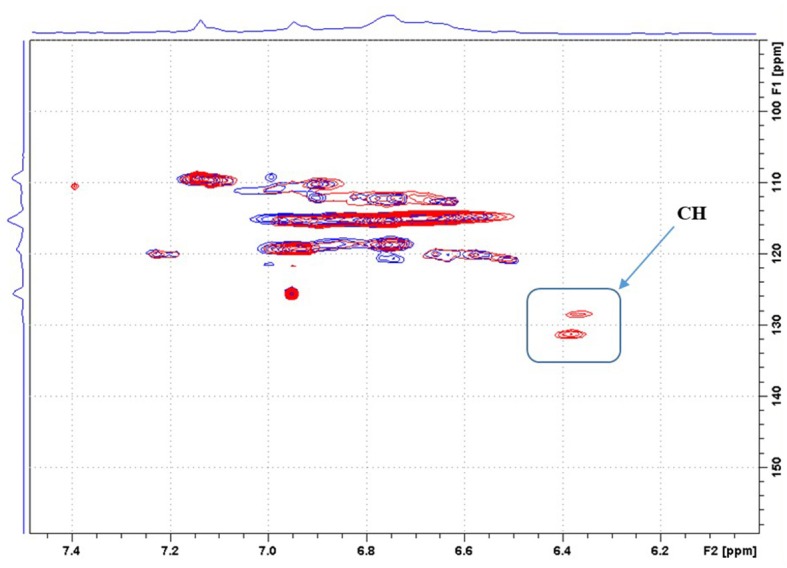
HSQC spectrum of native KL and KL0.2MA in DMSO, aromatic zone.

The disperse and complex structure of lignin makes ^1^H and ^13^C NMR analysis very difficult. On the other hand, 2D NMR spectroscopy yields spectra that provide more useful information. HSQC analyses of lignins (in DMSO-*d*_6_) were performed. The signal of DMSO was set at 2.5 ppm for ^1^H and 39.5 ppm for ^13^C. The HSQC spectra of native KL and KL modified with 0.2 MA were compared. As can be seen in the enlargement of this spectrum (Figure 2: blue signals for native KL CH or CH_3_ and red/pink signals for KL 0.2MA CH or CH_3_/CH_2_), two new spots appeared at (1) 6.37 ppm (^1^H); 128.7 ppm (^13^C) and (2) 6.39 (^1^H); 131.3 (^13^C). These signals are attributed to the CH groups of the grafted maleic anhydride. These signals are absent for the native KL. In addition, they do not correspond to the signals of MA or maleic acid that could be formed during the washing of samples with water. The appearance of two spots and not only one is due to the different chemical environment of these two CH groups: ester on one side and carboxylic acid on the other. This trend is observed for all samples (0.1; 0.2 and 0.3 MA). This confirms that the esterification reaction has occurred. In this case, the reaction is a mono-esterification. This result is in good agreement with the elemental and FT-IR analysis.

**Figure 3:**
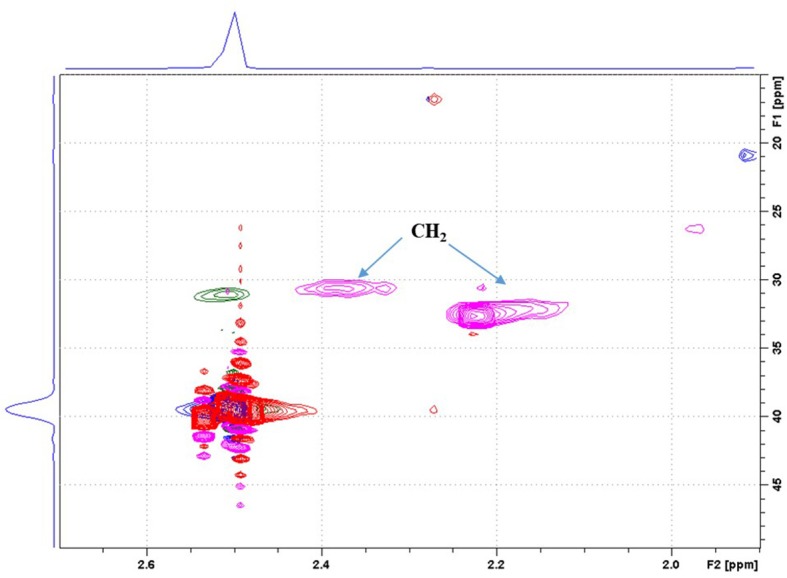
HSQC spectrum of native KL and KL0.2SA in DMSO, aliphatic zone.

In the same manner, the HSQC spectra of native and succinic anhydride modified lignin 0.2SA KL were superimposed (Figure 3) -blue/green signals: CH or CH_3_/CH_2_ for the native KL; Red/pink signals: CH or CH_3_/CH_2_ for KL0.2SA-. As in the case of MA, the HSQC enlargement of SA modified KL confirms the appearance of two new signals (pink) corresponding to CH_2_ groups with different chemical environment. Full HSQC spectra for KL0.2MA and KL0.2SA are available in Supporting Information (Figures S3, S4).

### Phosphorus NMR Analysis

The obtained proportions of the different hydroxyl and carboxylic acid groups (in mmol/g) for both MA and SA modified KL are summarized in Table 2. For both anhydrides, the total amount of hydroxyl decreases during the reaction. After esterification, the hydroxyl content decreased by 34% (KL0.3MA). This also confirms that the esterification has taken place. The carboxylic acid content increased after esterification. Aliphatic and aromatic hydroxyls show a different reactivity. A 48% decrease was observed for the aliphatic hydroxyl content while only 23% of the phenolic hydroxyl reacted. Therefore, esterification was more effective on aliphatic hydroxyl groups than on phenolic hydroxyl groups. This trend was also observed by Ahvazi et al. (2011) during the esterification of kraft lignin with MA in batch. On the other hand, Bridson et al. (2013) described the possible decrease in aliphatic hydroxyl and carboxylic acids after extrusion of kraft lignin without plasticizer. According to them, the decrease in hydroxyl levels is probably due to dehydration reactions leading to condensation. These results highlight condensation reactions when using reactive extrusion for lignin chemical modification. Similar results can be observed for the esterification reaction with succinic anhydride. The percentage of hydroxyl decreases after esterification, with the increase in the amount of carboxylic acid. Aliphatic OHs are also more reactive than aromatic OHs. The sample KL0.3SA was not soluble in the used solvent system. The reference sample (KL_ref_) showed a decrease in both aromatic and aliphatic OHs as well as carboxyl groups. Phenolic OH groups decreased from 1.81 mmol/g for native KL to 1.42 mmol/g. Aliphatic OHs decreased from 1.54 mmol/g (native KL) to 1.39 mmol/g (KL_ref_). This result reveals that the structure of lignin changes during the REX process even when no reagent is used. In contrast, the samples of KL modified with MA and SA prepared in batch conditions show very poor solubility in pyridine/deuterated chloroform mixture used for ^31^P NMR, which might further indicate (along with the increase in molecular weight) a possible crosslinking between free carboxyl groups of grafted anhydride and the residual hydroxyl groups still present in lignin.

**Table 2:** Proportion in mmol/g of lignin, of the different hydroxyl groups and carboxylic acids of native and esterified KL.

**KL**	**COOH**	**G-OH**	**S-OH**	**H-OH**	**Total phenolic-OH**	**Total aliphatic-OH**	**Total hydroxyl**
Native	0.22	1.43	0.23	0.15	1.81	1.54	3.35
0.1 MA	0.28	1.31	0.18	0.12	1.61	1.3	2.91
0.2 MA	0.34	1.25	0.14	0.1	1.49	1.15	2.64
0.3 MA	0.56	1.2	0.12	0.08	1.40	0.8	2.2
0.1 SA	0.27	1.32	0.2	0.1	1.62	1.25	2.87
0.2 SA	0.42	1.29	0.12	0.08	1.49	1.2	2.69

### Solid-State ^13^C CP-MAS NMR

**Figure 4:**
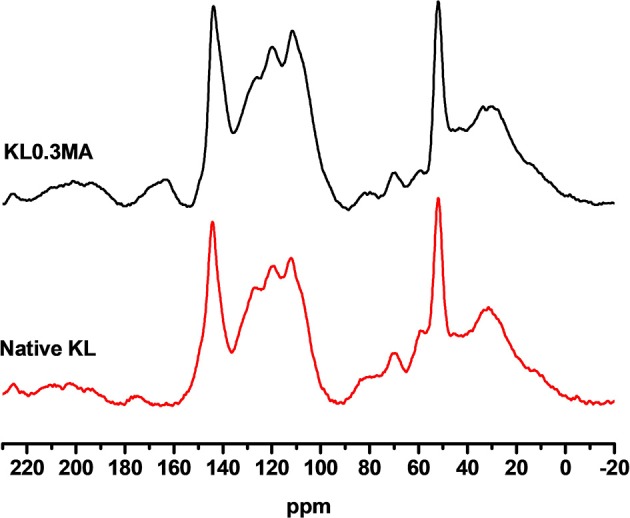
Solid-state ^13^C CP-MAS NMR spectra of Native KL and KL modified with 0.3 equivalents of MA.

It was not possible to calculate the DS of esterified lignin samples using ^31^P NMR due to possible dehydration involving hydroxyl groups of lignin. On the other hand, Solid-state ^13^C CP-MAS NMR allows us to calculate the DS using the signals of grafted carbonyl groups after esterification. Figure 4 shows solid-state ^13^C CP-MAS NMR spectra of native and modified (KL0.3MA) lignins. It can be seen that after esterification with maleic anhydride the carbonyl group (175 ppm) which is present in native lignin in small quantity increases significantly and shifts to higher field (163 ppm). This shift can be explained by the difference in the chemical environment of carbonyl functions in native and esterified lignins. The DS of modified sample (KL0.3MA) was found to be 0.18 with a corresponding RE of 60%. The Solid-state ^13^C CP-MAS NMR spectrum of batch modified KL (Batch KL0.3MA) is shown in Supporting Information (Figure S5). The DS of this sample is 0.19 with RE of 63%.

### GPC/SEC Chromatography

**Figure 5:**
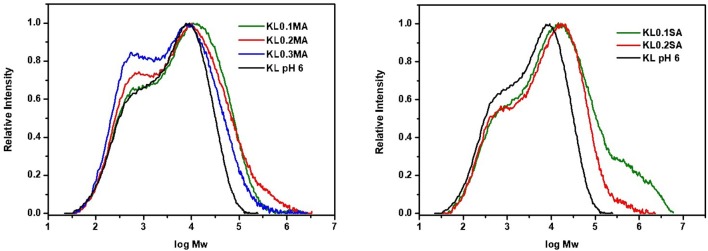
GPC/SEC chromatograms of REX modified KL with maleic **(Left)** and succinic **(Right)** anhydrides.

**Figure 6:**
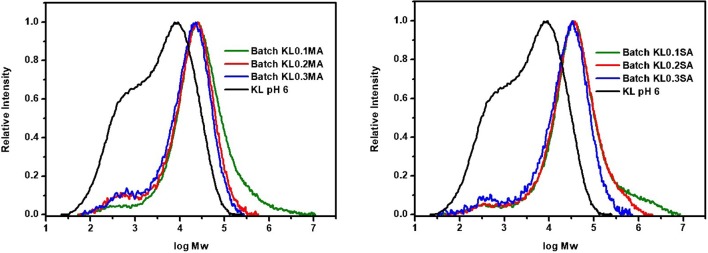
GPC/SEC chromatograms of KL modified in batch with maleic **(Left)** and succinic **(Right)** anhydrides.

Chemical modification of lignin in REX conditions, where shear rate and temperature play essential role, can have a direct influence on molecular weight distribution. It was found that for native lignin M_w_ is 10,800 g/mol with a polydispersity of 8.91. As we can see in Figure 5, after extrusion the polydispersity increases significantly for both MA and SA lignin esters. However, the chromatograms are different for two reagents. In the case of KL modified with MA, a new peak with M_w_ = 560 g/mol appeared in the area of low molecular weight fractions, which increases with increasing ratio of the anhydride. This can be explained by possible lignin chain cleavage during REX conditions (Bridson et al., 2013). The original peak of lignin becomes broader after the reaction. For KL modified with SA, there are no changes in low molecular weight area. At the same time, the original peak is shifted toward higher molecular weight showing higher polydispersity than native KL. These results show that REX can change the structure and properties of lignin. In contrast, KL esterified in batch conditions (Figure 6) shows a shift of molecular weight profiles toward higher molecular weights for both anhydrides. In this case, one possible explanation could be intra- and intermolecular crosslinking between remaining hydroxyls of lignin and free carboxyl groups newly generated after the reaction with the anhydride. As previously mentioned (section Phosphorus NMR analysis), the KL samples esterified in batch show poor solubility in ^31^P NMR solvent which also indicates the possible crosslinking in the lignin polymer. The crosslinking has an influence not only on lignin molecule but also on the properties of lignin-based materials (Lang et al., 2018). However, the polydispersity decreases in the case of reaction in batch from 8.91 for native KL to 6 for Batch KL0.3MA and 7.82 in case of Batch KL0.3SA. This result could be also due to the purification procedure, with the low molecular weight fractions being more soluble in acetone. To highlight the influence of REX parameters on the lignin transformation, the reference sample KL_ref_ was studied by GPC/SEC. It was found that after extrusion, the molecular weight and polydispersity of lignin increase (M_w_ = 15900, M_w_/M_n_ = 11). This result confirms that temperature and shear rate have a significant influence on lignin structural changes. The future interest in lignin transformation using REX would be to explore if these changes are directly correlated to SME and if it is possible to control the average molecular weight and polydispersity of the obtained product by the extrusion process parameters.

### Thermal Analysis of Native and Modified KL

**Figure 7:**
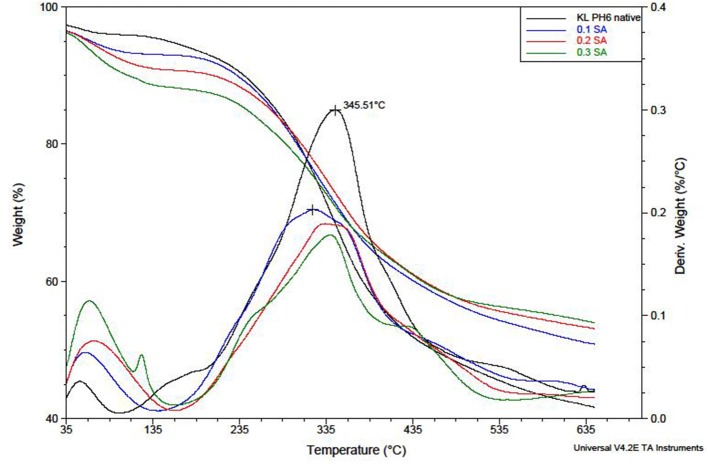
TGA and DTG curves (recorded under nitrogen atmosphere) of native kraft lignin and kraft lignin esterified with SA.

Native and modified KL were analyzed using TGA (Figure 7). For all samples, a weight loss of about 5–8% at temperatures up to 100°C corresponds to water loss. The water loss percentage increases with the increase of succinic anhydride. This result confirms that modified samples become more hydrophilic. During additional heating, the onset of degradation occurred between 135 and 200°C. Typically, the degradation rate reaches a maximum between 300 and 400°C. During this stage (up to 600°C) the pyrolysis of the lignin takes place, resulting in the breaking of the C-C and C-O bonds of the side chains, the condensed bonds and the breaking of the ether bonds (Cao et al., 2013). From 600°C, the formation of amorphous carbon residue is observed. The samples of KL modified with succinic anhydride all show a slower degradation compared to native lignin. The same behavior is observed in the case of MA. However, no significant changes in thermal behavior between native KL and the reference sample are observed.

## Conclusion

In summary, we found that kraft lignin maleic and succinic esters can be prepared in very short reaction time (<1 min) in a homogeneous medium and by using low amounts of plasticizer (15–25%) in a reactive extrusion process. Native lignin shows no melting behavior. We showed that small amounts of DMSO renders lignin processable when low extrusion temperature (140°C) is applied. 2D NMR spectroscopy revealed that the reaction of lignin transformation via REX is a monoesterification. At the same time, the molecular weight and polydispersity increase in high shear rate + temperature REX conditions resulting in lignin molecules crosslinking. GPC chromatograms after extrusion showed a shift toward higher molecular weights. A similar shift was also observed for samples prepared in batch conditions. In contrast, the polydispersity decreases for lignin esters synthesized in batch. REX esterified lignin samples show slower degradation compared to native lignins. These new lignin derivatives could be used in combination with other polymers to produce new plastics. Due to the presence of a pendant reactive double bond maleated lignin could be used as a macromonomer for further polymerization or copolymerization synthesis.

## Data Availability

All datasets generated for this study are included in the manuscript/Supplementary Files.

## Author Contributions

All the authors have contributed to the work presented in the manuscript to an extent that is consistent with the criteria for authorship. All the authors have agreed with the contents.

### Conflict of Interest Statement

The authors declare that the research was conducted in the absence of any commercial or financial relationships that could be construed as a potential conflict of interest.
