# Supplementary material for: Chemical Modification of Plasticized Lignins Using Reactive Extrusion
Source: Front Chem. 2019 Sep 18;7:633. doi: 10.3389/fchem.2019.00633 (PMC6759957; doi:10.3389/fchem.2019.00633)
Supplement: Supplementary file 1 [file Data_Sheet_1.PDF]

Chemical Modification of Plasticized Lignins Using Reactive Extrusion

Romain Milotskyi [a,b*], László Szabó [b], Kenji Takahashi [b], Christophe Bliard [a]

[a]Institut de Chimie Moléculaire de Reims, ICMR, CNRS UMR 7312, URCA, B18, UFR SEN, Moulin de la Housse, Chemin des Roulliers, BP 1039, Reims, Cedex 2, F 51 687, France

[b]Institute of Science and Engineering, Kanazawa University, Kakuma-machi, Kanazawa 920-1192, Ishikawa, Japan

* Correspondence

Corresponding Authors:

romain-mi@se.kanazawa-u.ac.jp; szabo-laszlo@se.kanazawa-u.ac.jp; ktkenji@staff.kanazawa-u.ac.jp; christophe.bliard@univ-reims.fr

Key words: Plasticizing, kraft lignin, esterification, ^{31}P NMR, DMSO, polyol

Supporting information

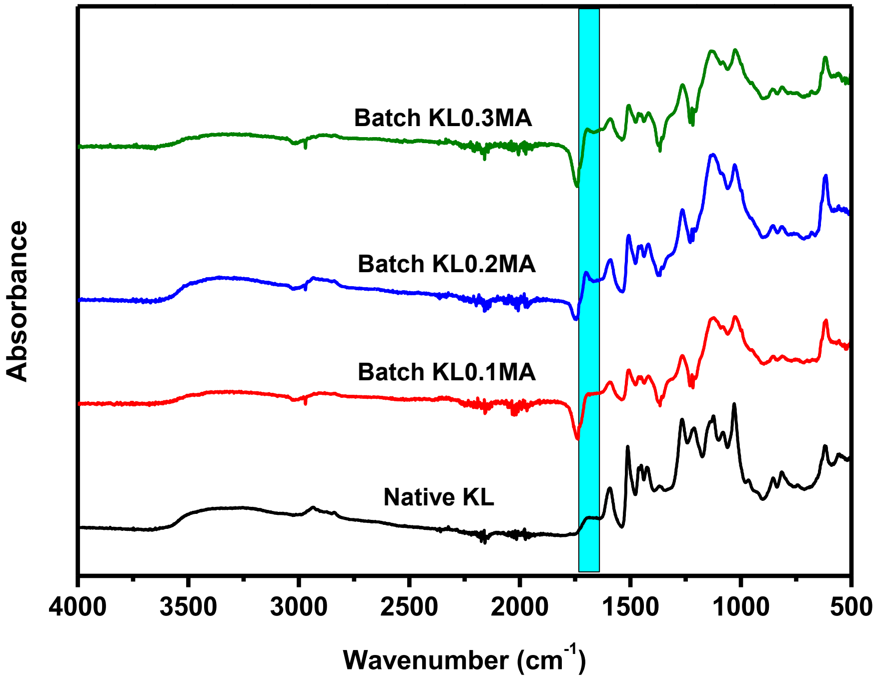

Figure S1: ATR spectra of KL modified with different ratio of maleic anhydride in batch conditions

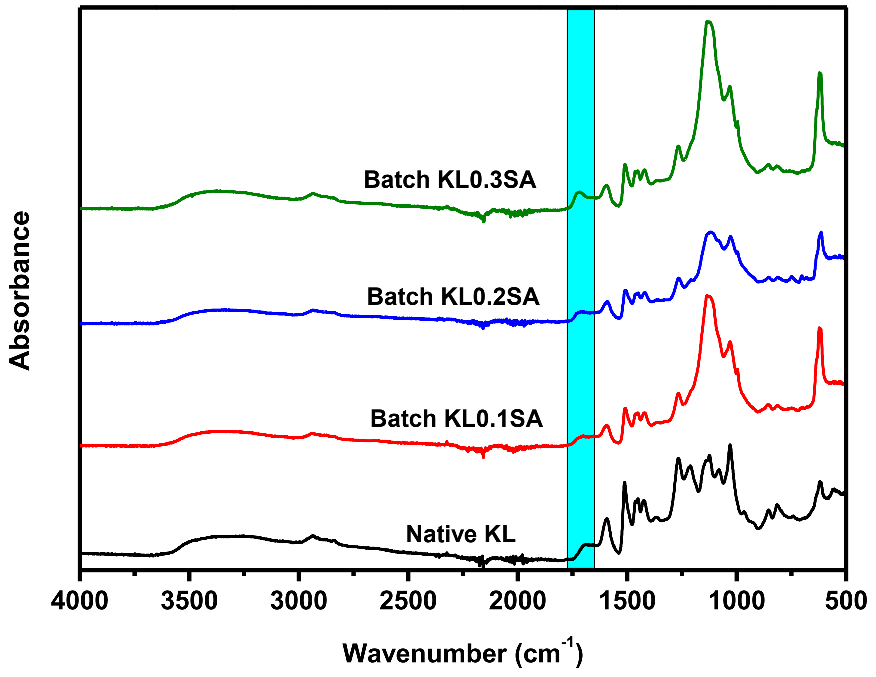

Figure S2: ATR spectra of KL modified with different ratio of succinic anhydride in batch conditions

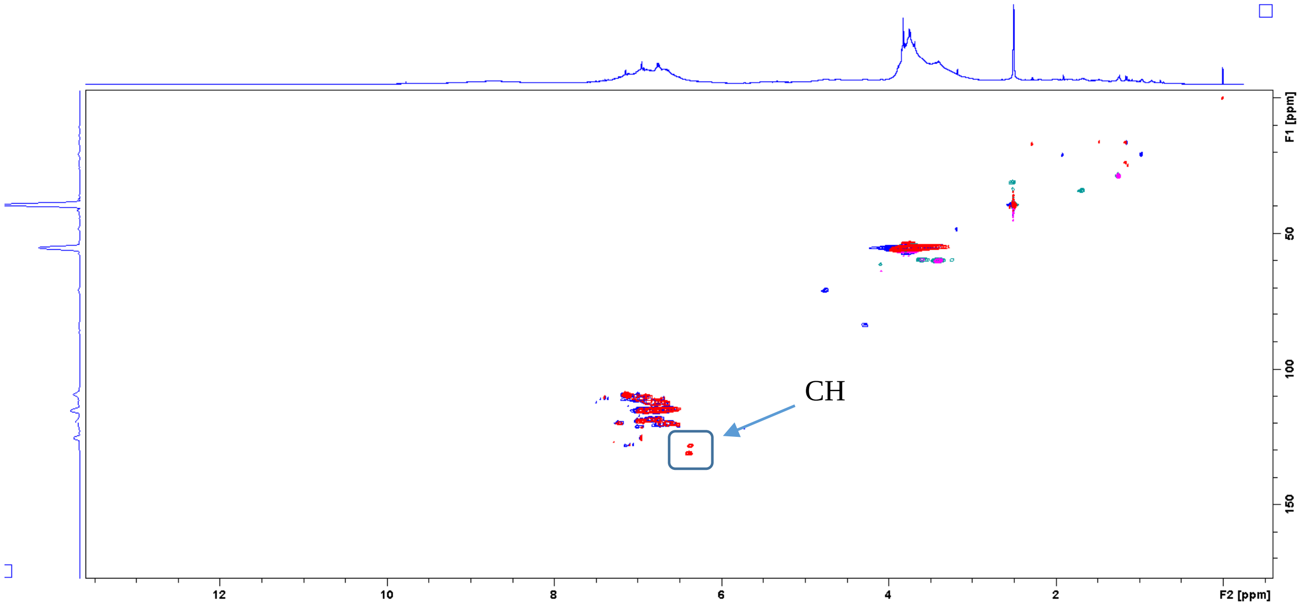

Figure S3: HSQC spectrum of native KL and KL0.2MA in DMSO

Blue/green signals: CH or CH3 / CH2 from the native KL; Red/pink signals: CH or CH3 / CH2 from KL 0.2MA

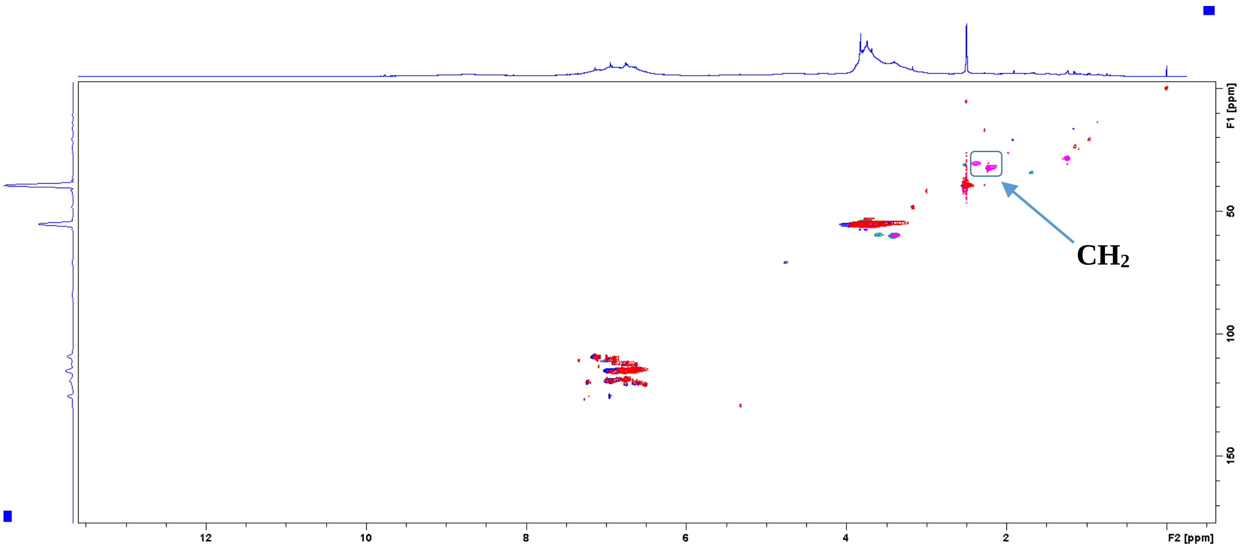

Figure S4: HSQC spectrum of native KL and KL0.2SA in DMSO

Blue/green signals: CH or CH3 / CH2 from the native KL; Red/pink signals: CH or CH3 / CH2 from KL0.2SA

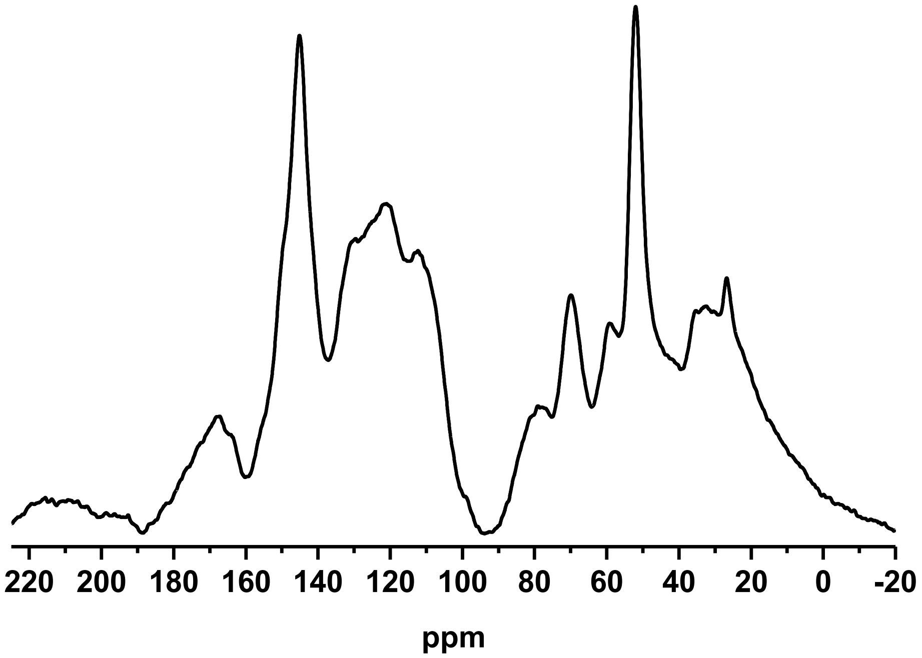

Figure S5: Solid-state ^{13}C CP-MAS NMR spectra of KL modified with 0.3 equivalents of MA in batch conditions (Batch KL0.3MA)
